# Grafting of Black Pepper onto Native *Piper* Species

**DOI:** 10.3390/plants15203133

**Published:** 2026-10-14

**Authors:** Basílio Cerri Neto, Vinicius de Souza Oliveira, Johnatan Jair de Paula Marchiori, Jeane Crasque, Thayanne Rangel Ferreira, Simone Alves Fernandes, Carla da Silva Dias, Lúcio de Oliveira Arantes, Elmo Pereira Ramos, Antelmo Ralph Falqueto, Edilson Romais Schmildt, Sara Dousseau-Arantes

**Affiliations:** 1 Departamento de Ciências Biológicas, Centro de Ciências Humanas e Naturais, Universidade Federal do Espírito Santo, Avenida Fernando Ferrari, 514, Goiabeiras, Vitória 29075-910, ES, Brazil; basiliocerri@yahoo.com.br (B.C.N.); jeanecrasker@gmail.com (J.C.); thayannerangel85@gmail.com (T.R.F.); 2 Centro de Pesquisa, Desenvolvimento e Inovação Norte, Instituto Capixaba de Pesquisa, Assistência Técnica e Extensão Rural, Caixa Postal 62, Linhares 29900-970, ES, Brazil; johnatanmarchiori@gmail.com (J.J.d.P.M.); carla.dias@incaper.es.gov.br (C.d.S.D.); lucio.arantes@incaper.es.gov.br (L.d.O.A.); elmoepr@gmail.com (E.P.R.); 3 Departamento de Ciências Agrárias e Biológicas, Centro Universitário Norte do Espírito Santo, Universidade Federal do Espírito Santo, BR101 Norte, Km. 60, Bairro Litorâneo, São Mateus 29932-540, ES, Brazil; fernandespiont@gmail.com (S.A.F.); antelmofalqueto@gmail.com (A.R.F.); e.romais.s@gmail.com (E.R.S.); 4 Homeoplant, Pinheiros 29980-000, ES, Brazil

**Keywords:** *Piper aduncum*, *Piper caldense*, *Piper nigrum*, compatibility, resistance, *Fusarium*

## Abstract

Brazil is the third largest producer of black pepper in the world. Although Brazil is prominent in black pepper production, the average yield is low. The main stressful factors in Brazilian black pepper crops directly affect the root system, such as water deficit, flooding and diseases. One strategy to enable the cultivation of black pepper in contaminated areas is the planting of seedlings grafted onto wild Piper species resistant to fusariosis, drought or flooding. Grafting consists of the union of plant tissues from at least two different plants to generate a single organism. However, many factors influence graft incompatibility, the main ones being botanical affinity, which is directly linked to genetic and morphological factors, environmental conditions and physiological factors. The objective of this work was to investigate the compatibility and physiology of grafted seedlings of ‘bragantina’ black pepper, using three rootstocks. The experiment was conducted at the Linhares Experimental Farm, of the Capixaba Institute for Research, Technical Assistance and Rural Extension. *Piper aduncum*, *Piper caldense* and the cultivar bragantina (*Piper nigrum*) were evaluated as rootstock. The native species were propagated by seeds and the bragantina cultivar by cuttings, both propagules collected from mother plants in the Active Germplasm Bank located on the farm. The full cleft top method was carried out. The rootstocks were decapitated just below the fourth node, in which a slit was made, 3 cm deep, and after insertion of the graft, it was tied with a rubber band. The grafts were covered with plastic bags. After grafting, the plants were kept in a nursery. Four months after grafting, survival, chlorophyll *a* fluorescence, gas exchange, hydraulic conductivity of the stem and root, shoot and root anatomy were assessed. At the end of the experiment, the survival rate was higher in plants grafted onto *Piper caldense. P. aduncum* showed an increase in the number of active FSII reaction centers by cross sections (RC/CS0). The photosynthetic performance index relative to absorption (PIabs) was increased in *P. aduncum*. *P. caldense* and bragantina showed no significant difference. Water use efficiency (WUE) was higher in *P. caldense* plants, followed by bragantina plants. *P. aduncum* showed a decrease in WUE. *P. aduncum* showed an increase in the length of the vascular bundle and root xylem, however *P. caldense* showed reduced values. Regarding the number of root xylem vessels, the highest values were observed in bragantina plants. The diameter of the root xylem vessels was higher in *P. aduncum*. *P. caldense* and bragantina showed no difference. It is concluded that among the species studied, *P. caldense* was the species that presented the most characteristics that make it compatible with being used as a rootstock using the bragantina genotype as a graft.

## 1. Introduction

Species of the genus *Piper* are of global economic importance due to phytochemicals found throughout the plant, which are used to produce pharmaceuticals, cosmetics, foods and agricultural products ranging from crop protection agents to biostimulants [1]. Black pepper (*Piper nigrum* L.) is the most economically important cultivated species within the genus. Its fruits are considered the world’s most popular condiment, earning the title ‘King of Spice’ [2].

Black pepper is a perennial climbing plant native to India; it was introduced to Brazil in 1930, though its cultivation began to spread in 1933 [3]. Brazil is the world’s third-largest producer, according to data from the Food and Agriculture Organization of the United Nations (FAO) [4]. Brazilian production is concentrated in just two states: in 2020, Espírito Santo accounted for 60% of production and Pará for 32%, despite having similar planted areas. Espírito Santo is a national leader in black pepper cultivation, achieving a yield of 4 t ha^−1^ [5].

Although Brazil is prominent in black pepper production, the average yield is 3 t ha^−1^, half the value achieved by Cambodia and Costa Rica and around 85% lower than Tajikistan, where average yields of 21 t ha^−1^ were reported [4]. These data highlight the low national productivity, but at the same time it exposes the potential for improvement and the need to develop technologies to be incorporated into production systems. Environmental stresses of a biotic and abiotic nature reduce the productivity of black pepper throughout the world [6].

The main stressful factors in Brazilian black pepper crops directly affect the root system, such as water deficit, flooding and diseases. Soil diseases restrict the productivity and longevity of black pepper crops around the world, particularly fusariosis, nematodes and phytophtora. However, fusariosis, caused by the fungus *Fusarium solani* f. sp. Piperis, endemic to Brazil, damages the root system and can lead to plant death [7].

The fungus causes root rot by colonizing the xylem vessels, thereby disrupting the flow of water and nutrients within the plant. Additionally, the plant may exhibit defense responses against the pathogen such as the formation of tyloses or the presence of lignolytic and cellulolytic enzymes employed to protect itself from the pathogen’s toxins; consequently, these mechanisms impede the transport of water and nutrients [8].

Once the pathogen infects the plant, farmers attempt to control the disease using fungicides; however, this is not a low-cost option due to the need for constant application [9]. Furthermore, only inorganic copper-based products are registered with MAPA for managing fungal and bacterial diseases in Brazilian black pepper crops, severely limiting both technical recommendations for farmers and the overall efficacy of control measures.

One strategy that could make black pepper cultivation feasible in contaminated areas is planting seedlings grafted onto wild *Piper* species resistant to *Fusarium* wilt. Albuquerque et al. [10] note that certain native species (*Piper tuberculatum* Jacq., *Piper aduncum* L., *Piper arboreum* Aubl. and *Piper colubrinum* Link.) exhibit resistance to root pathogens, identifying them as promising rootstocks for black pepper. However, given that there are 289 native *Piper* species in Brazil, many others may also be resistant to *Fusarium* wilt.

Grafting involves joining the plant tissues of at least two different plants to create a single organism [11]. Grafting has long been successfully applied in horticulture and agriculture for purposes such as improving plant vigor, increasing resistance to diseases and stress, and plant propagation. Initially established for woody plant species, the practice has more recently expanded to include various herbaceous species. Thanks to the development of new graft combinations and automation techniques, over a billion plants are grafted worldwide today, with the number increasing annually [12].

However, many factors influence graft incompatibility, the main ones being botanical affinity, which is directly linked to genetic and morphological factors, environmental conditions and physiological factors [11]. Incompatibility can occur in two ways, early, that is, the grafted plant shows signs of death or rejection within a few days or even hours after grafting. However, late incompatibility can occur between three and five years after grafting [13].

Early incompatibility is observed mainly when there is a vigorous rootstock and a less vigorous graft, which causes vigorous budding of the bud, in addition to elongation of the stem, thus preventing the connection between the tissues, followed by the death of the plant [14].

On the other hand, it is known that grafting is a complex propagation process that depends on the interaction between graft and rootstock, in addition to grafting techniques and environmental conditions. Studies on the factors that determine the success of grafting in species of agronomic interest have advanced significantly in recent years; however, the same has not occurred with black pepper. The objective of this work was to investigate the compatibility and physiology of grafted seedlings of ‘bragantina’ black pepper, using three rootstocks.

## 2. Results

### 2.1. Survival

**Figure 1:**
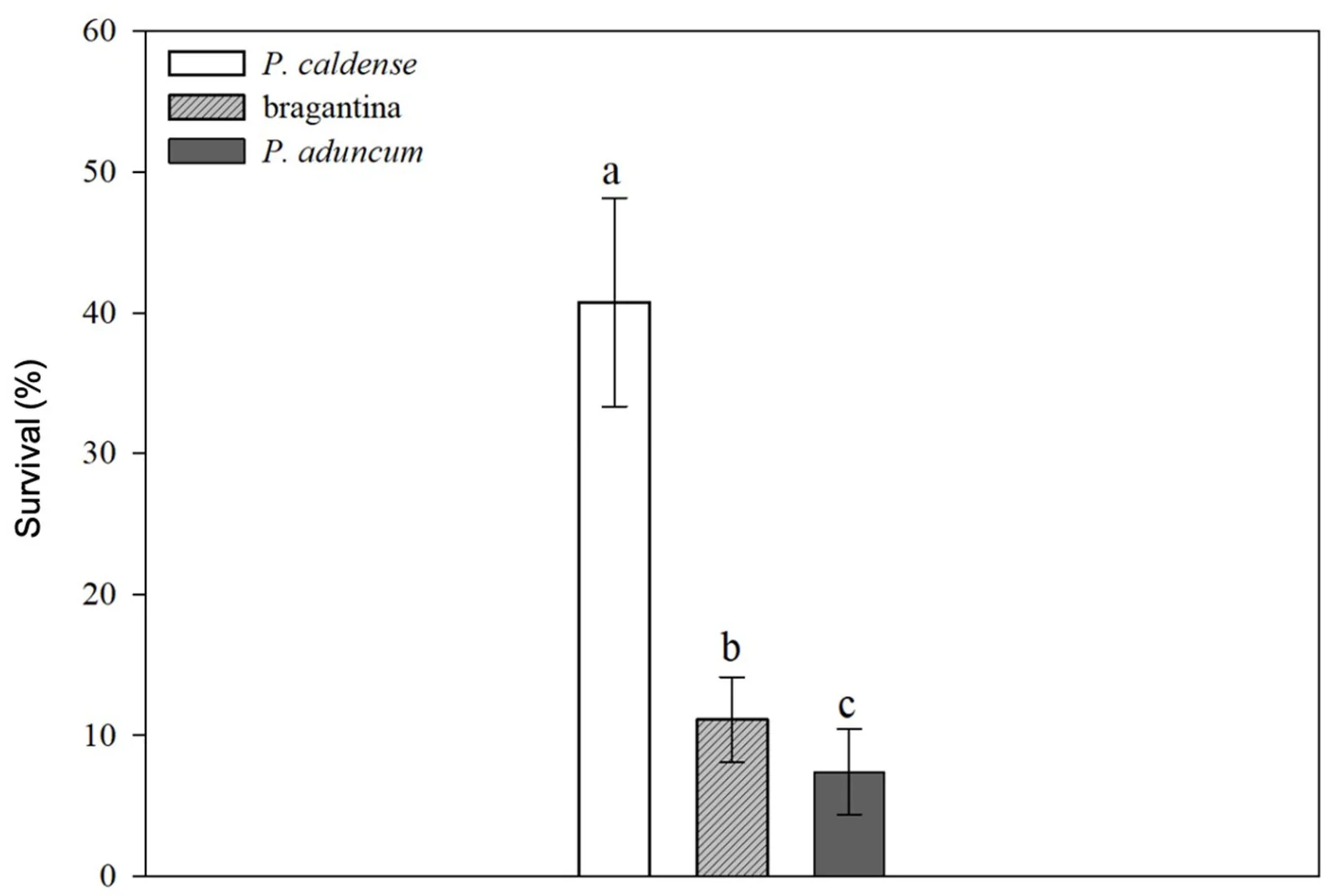
Survival percentage of bragantina black pepper under different rootstocks, *Piper aduncum*, *Piper caldense* and bragantina. Means followed by the same letter do not differ from each other using the Scott-Knott test at the 5% probability level. The bar corresponds to the standard error of the mean of four repetitions.

At the end of the experiment, the survival rate was higher for plants grafted onto *Piper caldense* (38.88%), followed by bragantina (11.11%), while *Piper aduncum* showed the lowest survival rate (7.41%) (Figure 1). Therefore, out of the 108 plants evaluated for each rootstock (27 from each of the four blocks), approximately 42 *Piper caldense*, 12 bragantina and 8 *Piper aduncum* plants survived.

### 2.2. Chlorophyll a Fluorescence

The maximum photochemical quantum yield (φP0) and the quantum yield of electron transfer from QA to the electron transport chain beyond QA (φE0) were statistically higher in *P. aduncum* plants compared to plants grafted onto *P. caldense* and bragantina (Table 1).

*P. caldense* and bragantina showed an increase in the energy absorption flux per reaction center (ABS/RC), energy captured flux per reaction center (TR0/RC), the energy dissipated flux per reaction center (DI0/RC) and the photochemical quantum yield for heat dissipation (φD0); however, *P. aduncum* showed a decrease (Table 1). *P. aduncum* showed an increase in the number of active FSII reaction centers per cross sections (RC/CS0), *P. caldense* showed a considerable reduction in RC/CS0. The photosynthetic performance index relative to absorption (PIabs) was increased in *P. aduncum*. *P. caldense* and bragantina showed no significant difference (Table 1).

### 2.3. Gas Exchange

**Figure 2:**
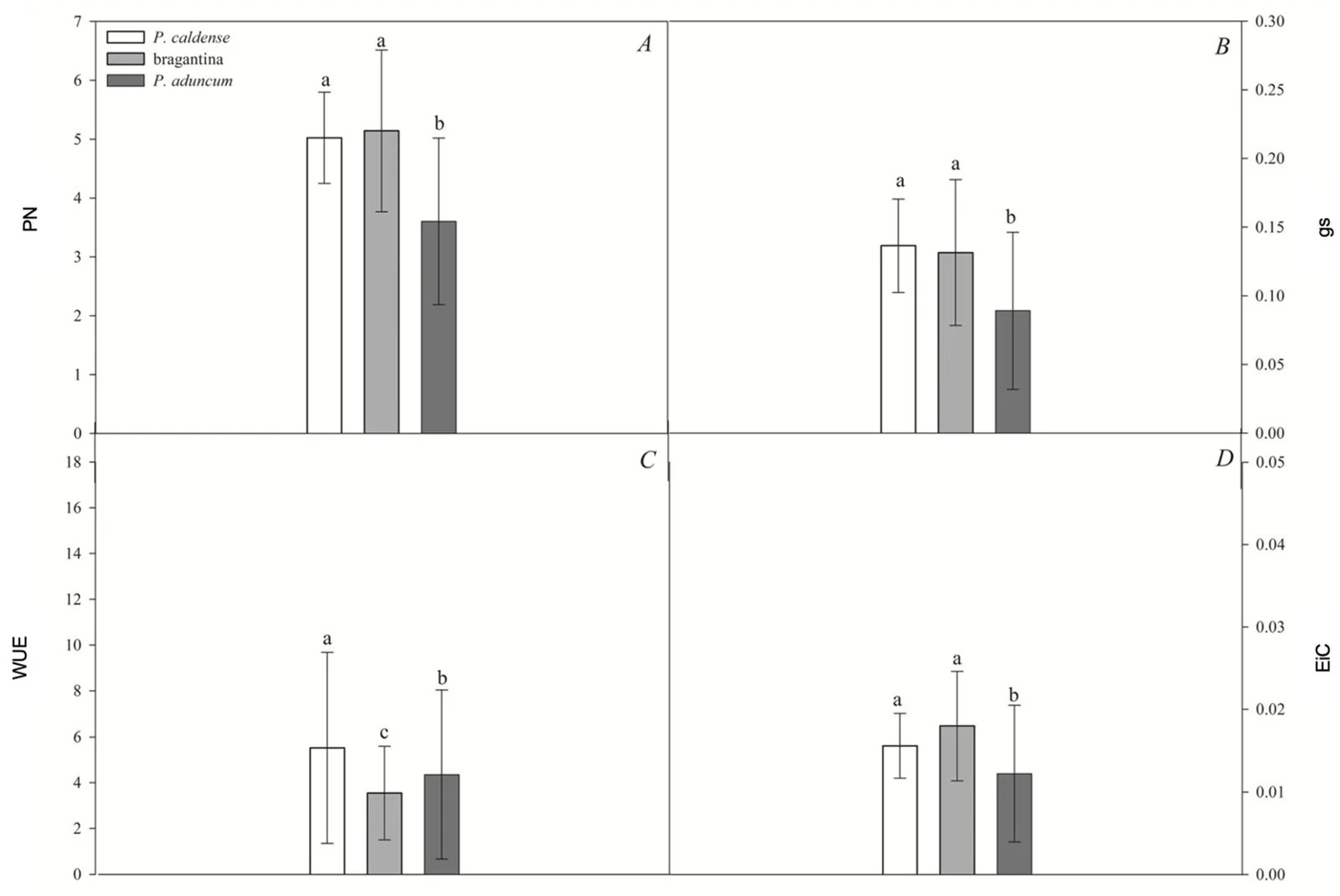
Gas exchange in bragantina black pepper seedlings under different rootstocks, *Piper aduncum*, *Piper caldense* and bragantina. Means followed by the same letter do not differ from each other using the Scott-Knott test at the 5% probability level. The bar corresponds to the standard error of the mean of four repetitions. (**A**) Photosynthetic rate (PN), (**B**) stomatal conductance (gs), (**C**) water use efficiency (WUE), (**D**) instantaneous carboxylation efficiency (EiC).

The photosynthesis rate (PN) and stomatal conductance (gs) were higher in *P. caldense* and bragantina plants, *P. aduncum* presented considerably low gs values (Figure 2A,B). Water use efficiency (WUE) was higher in *P. caldense* plants, followed by bragantina plants. *P. aduncum* showed a decrease in WUE. The instantaneous efficiency of carboxylation (EiC) was increased in *P. caldense* and bragantina plants (Figure 2C,D). Just like in the USA, *P. aduncum* also showed a decrease in EiC.

### 2.4. Stem and Root Hydraulic Conductivity

**Figure 3:**
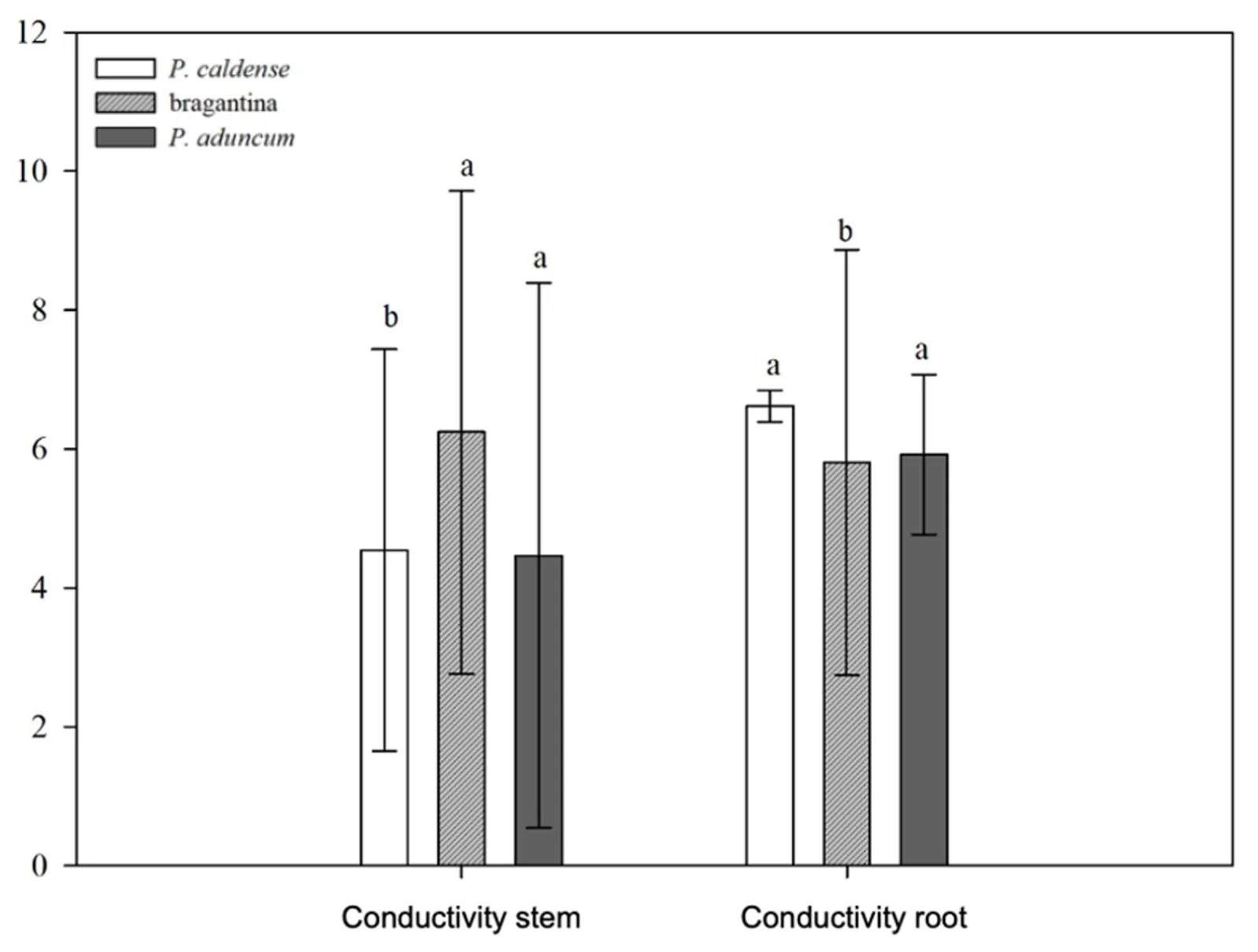
Hydraulic conductivity of the stem and root in bragantina black pepper seedlings under different rootstocks, *Piper aduncum*, *Piper caldense* and bragantina. Means followed by the same letter do not differ from each other using the Scott-Knott test at the 5% probability level. The bar corresponds to the standard error of the mean of four repetitions.

The hydraulic conductivity of the stem was reduced in *P. caldense* plants; however, there was a significant increase in *P. aduncum* and bragantina plants. However, root hydraulic conductivity was higher in *P. caldense* and *P. aduncum*. Bragantina showed a drastic reduction in root conductivity (Figure 3).

### 2.5. Anatomy

**Figure 4:**
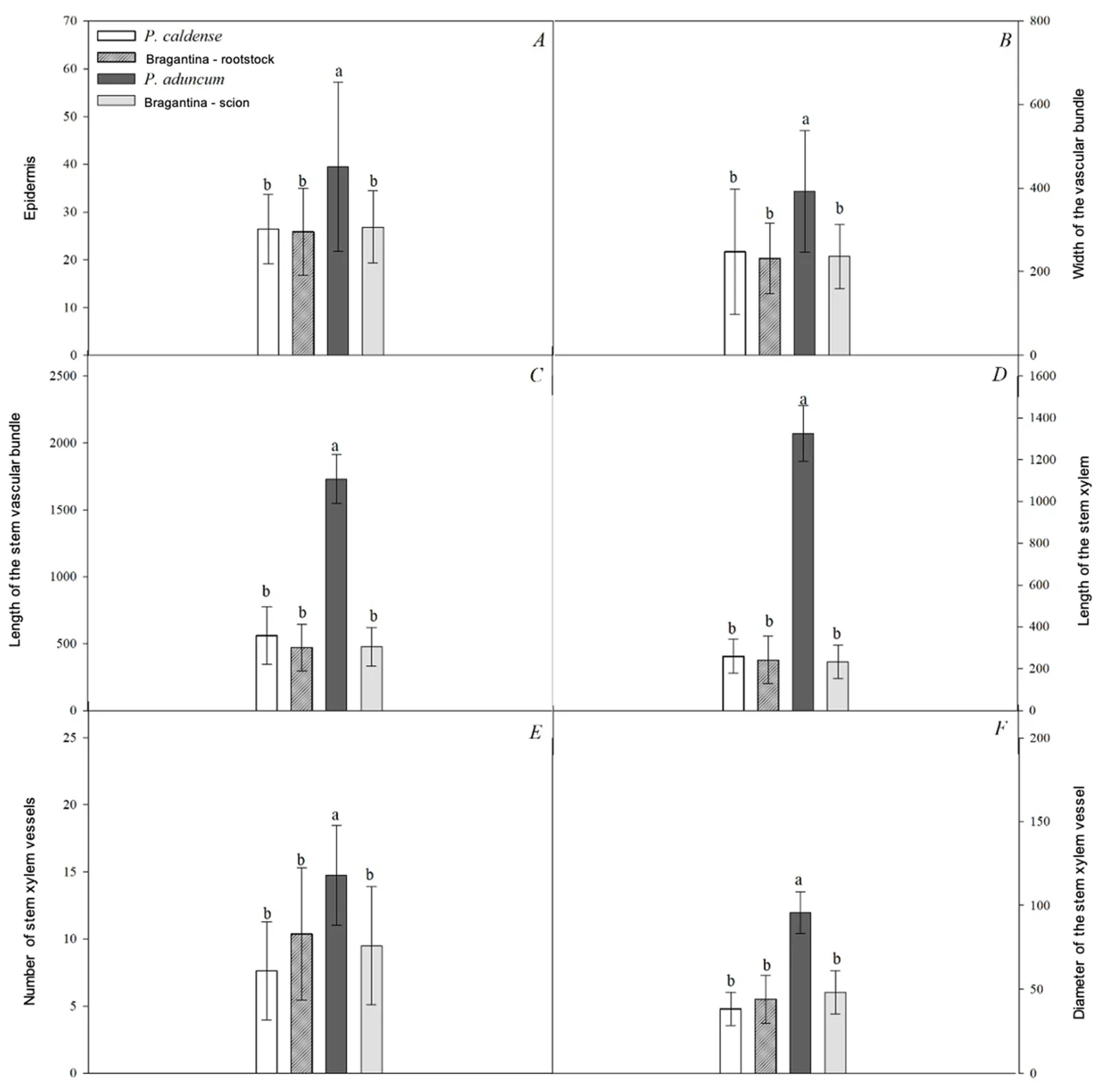
Stem anatomy in bragantina black pepper seedlings under different rootstocks, *Piper aduncum*, *Piper caldense* and bragantina. Means followed by the same letter do not differ from each other using the Scott-Knott test at the 5% probability level. The bar corresponds to the standard error of the mean of four repetitions. (**A**) Epidermis, (**B**) width of the vascular bundle, (**C**) length of the stem vascular bundle, (**D**) length of the stem xylem, (**E**) number of stem xylem vessels, (**F**) diameter of the stem xylem vessel.

Regarding stem anatomy, *P. aduncum* showed an increase in the epidermis, width and length of the vascular bundle, xylem length, number and diameter of xylem vessels, followed by *P. caldense* and bragantina graft and rootstock which showed no difference between them (Figure 4A–F).

**Figure 5:**
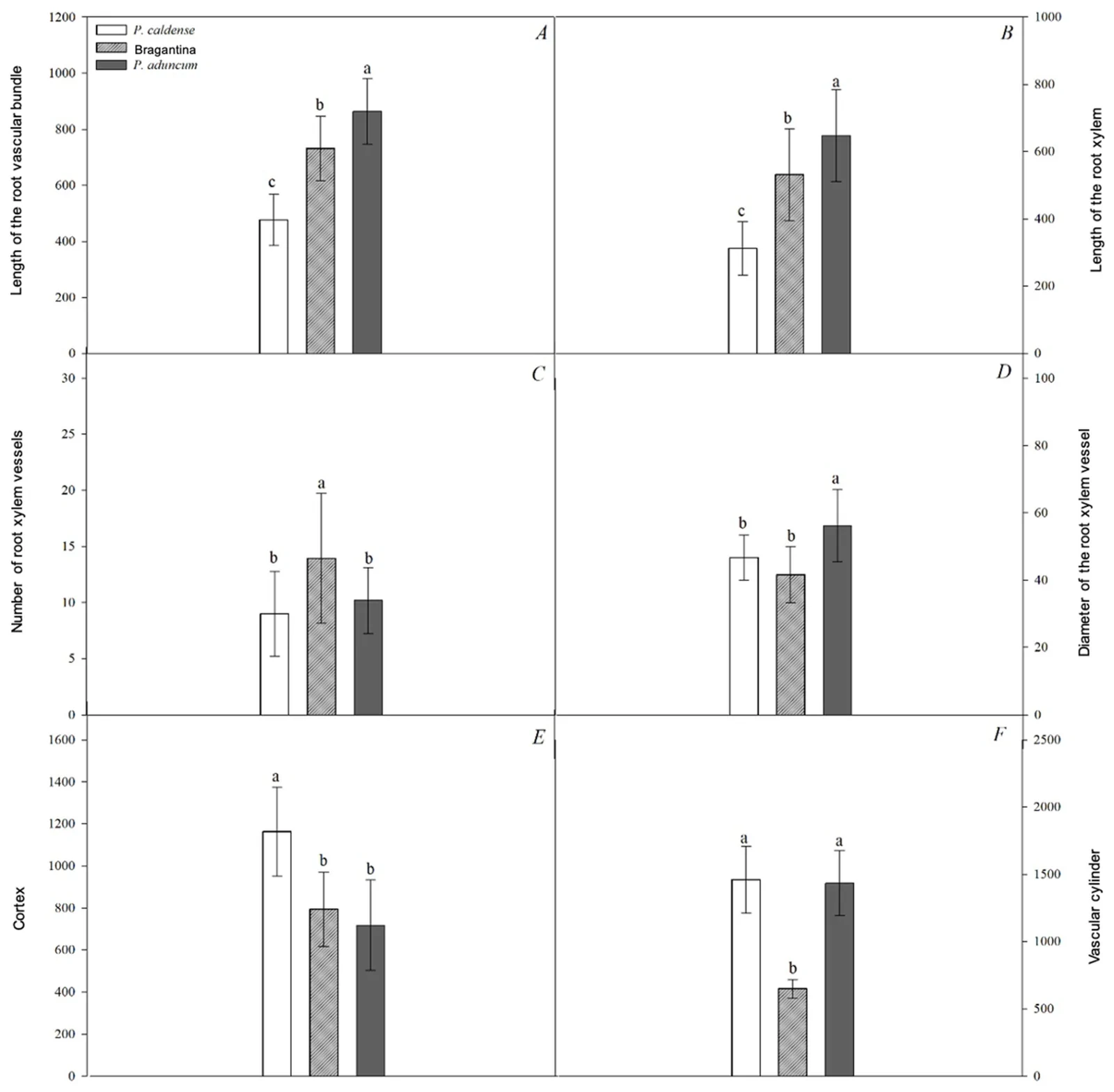
Root anatomy in bragantina black pepper seedlings under different rootstocks, *Piper aduncum*, *Piper caldense* and bragantina. Means followed by the same letter do not differ from each other using the Scott-Knott test at the 5% probability level. The bar corresponds to the standard error of the mean of four repetitions. (**A**) Length of the root vascular bundle, (**B**) length of the root xylem, (**C**) number of root xylem vessels, (**D**) diameter of the root xylem vessel, (**E**) cortex, (**F**) vascular cylinder.

*P. aduncum* showed an increase in the length of the vascular bundle and root xylem; however, *P. caldense* showed reduced values (Figure 5A,B). Regarding the number of root xylem vessels, the highest values were observed in bragantina plants. *P. aduncum* and *P. caldense* showed no significant difference between them (Figure 5C). The diameter of the root xylem vessels was higher in *P. aduncum*, *P. caldense* and bragantina showed no difference (Figure 5D). *P. caldense* showed greater cortex thickness, followed by *P. aduncum* and bragantina. Regarding the thickness of the vascular cylinder, an increase was observed in *P. caldense* and *P. aduncum*. Bragantina showed reduced values in the thickness of the cortex and vascular cylinder (Figure 5E,F).

## 3. Discussion

The success of the graft occurs when the plant presents a perfect junction of the tissues, caused by the uniformity of the material in terms of diameter, and an adequate welding of the tissues in the graft region, that is, callus formation, differentiated vascular bundle and connectivity at the graft interface, which guarantees balanced development [15].

Incompatible grafts at first present similar insertion behavior to compatible ones, but after days or months, the connection weakens and the graft is eliminated, while, in compatible combinations, the graft continues to develop [16]. Even though *P. aduncum* and bragantina showed a low percentage of survival in relation to *P. caldense*, both showed compatibility.

The maximum photochemical quantum yield (φP0) depicts the photochemical efficiency of photosystem II (PSII) [17]. The reduction in φP0 in *Piper caldense* and bragantina occurs because the efficiency in electron transport was compromised [18].

The reduction in the quantum yield of electron transfer from QA to the electron transport chain beyond QA (φE0) in *P. caldense* and bragantina grafted plants is a result of failure in electron transmission [19]. However, the increase in φE0 in *Piper aduncum* plants indicates that the graft did not compromise electron transmission.

The higher energy flux dissipated per reaction center (DI0/RC) and photochemical quantum yield for heat dissipation (φD0) in the grafted *P. caldense* and bragantina plants indicate a reduction in active reaction centers; these centers do not channel electrons to plastoquinone but instead dissipate them as heat, thereby preventing photoinhibition [20]. This increase is also associated with leaf protection against photo-oxidative damage [21]. In contrast, *P. aduncum* showed a decrease in DI0/RC and φD0 values, as the energy required to close all reaction centers and the energy dissipation flux per reaction center (DI0/RC) declined [22].

*P. caldense* and bragantina showed an increase in the energy absorption flux per reaction center (ABS/RC); this increase is caused by the relative size of the antenna connected to each reaction center changing [23]. However, the decrease observed in *P. aduncum* indicates a stress defense mechanism, which causes a change in the size of the FSII antenna system, which leads to the inactivation of a fraction of the reaction centers. Inactivation of reaction centers (reduction or heat dissipation centers) may be an indication of susceptibility to photoinhibition [20].

Despite having a higher survival rate, *P. caldense* and *P. bragantina* plants exhibited a reduction in the photosynthetic performance index based on absorption (PIabs) and the number of active PSII reaction centers per cross-section (RC/CS0) compared to *P. aduncum*. This reduction suggests a decline in overall photosynthetic performance associated with reduced electron transport capacity [20]. Reduced photosynthesis is common in plants under stress, given that grafting induces significant stress; however, despite the lower PIabs and RC/CS0 values, higher mean rates of photosynthesis (PN) and stomatal conductance (gs) were observed in *P. caldense* plants, indicating increased CO_2_ diffusion into the leaf mesophyll due to stomatal opening.

The instantaneous efficiency of carboxylation (EiC) is directly linked to the assimilation of carbon (CO_2_) where high values are associated with an increase in stomatal conductance (gs); this is observed in the species studied, where there is a significant increase in EiC in *P. caldense* and bragantina [24].

Much of the incompatibility observed between grafts occurs due to the growth and connections between tissues in the graft region, mainly the conduction tissues [14]. This causes damage to hydraulic conductivity and, as a consequence, the plant has difficulties in absorbing water and minerals, directly affecting growth and processes linked to photosynthesis. Throughout the plant growth process, there is a change in hydraulic conductivity governed by the evaporative demand of the aerial part, directly affecting the amount of water transported by the conductive tissue [25].

All stem anatomical structures of *P. aduncum* showed statistically significant differences compared to *P. caldense*, the ‘bragantina’ scion, and the ‘bragantina’ rootstock. This finding is significant because it demonstrates that the anatomical similarities observed among *P. caldense*, the ‘bragantina’ scion and the ‘bragantina’ rootstock facilitated the survival and establishment of the grafted plants—as evidenced by the higher survival rate of ‘bragantina’ plants grafted onto *P. caldense*. Thus, botanical affinity is crucial for grafting success, as the two plants involved must exhibit similar anatomical and cambial growth characteristics—as seen with *P. caldense* and ‘bragantina’. This is of utmost importance, given that cambial growth is a primary bottleneck in this propagation method [14].

The difference between the anatomical structures of the scion and the rootstock can be considered an important indicator of morphological incompatibility [26], which may be caused by differences in vigor between the scion and the rootstock, which can lead to a mismatch between the vascular systems in the grafting region and the development of the plant [27].

The rootstock is formed by a young plant, which can come from seeds or cuttings. These plants must be rustic, resistant to stress of biotic and abiotic origin. In addition, they must have good vegetative vigor, especially root system vigor and good development of anatomical structures [28]. The development of the xylem and xylem vessels of the root is extremely important for the success of the graft, considering that the rootstock will be responsible for meeting the water and nutritional needs of the graft [29]. However, the strength of the root anatomical structures must be similar to that of the graft; this reduces the chance of the graft not taking.

## 4. Materials and Methods

### 4.1. Plant Material and Cultivation Conditions

The experiment was conducted at the Linhares Experimental Farm, of the Capixaba Institute for Research, Technical Assistance and Rural Extension, located at 19°25′00.1″ S and 40°04′35.3″ W, in the municipality of Linhares, northern region of the state of Espírito Santo. *Piper aduncum*, *Piper caldense* and the cultivar bragantina (*Piper nigrum*) were evaluated as rootstock. The native species were propagated by seeds and the bragantina cultivar by cuttings, both propagules collected from mother plants in the Active Germplasm Bank located at FEL. The production and seedling formation stage took place from February to December 2020.

The seedlings of native species were obtained according to the protocol proposed by Dousseau et al. [30]. After 3 months of sowing, seedlings measuring 5 to 6 cm tall were selected and transplanted into tubes with a capacity of 280 cm^3^ filled with commercial organic substrate Bioplant (Minas Gerais, Brazil), containing 5 g per tube of slow-release fertilizer Osmocote^®️^(Rio Grande do Sul, Brazil), 6 months, formula NPK 15-09-12) and kept in a nursery under 50% shading obtained with black screens for another 4 months.

The seedlings of the black pepper cultivar bragantina were produced as described in Ambrozim et al. [31] with adaptations. Herbaceous cuttings with one node and two leaves were used, collected from the median region of the herbaceous branch. The cuttings were immersed in a solution containing Carbendazin fungicide for a period of 5 min. After this treatment, the cuttings were cut and placed in plastic pots containing a 400 mg.L^−1^ indole butyric acid growth regulator solution, so that only the basal part of the cuttings remained in contact with the solution for a period of 12 h. The cuttings were planted in tubes with a capacity of 450 cm^3^ filled with commercial organic substrate, fertilized with 5 g of slow-release fertilizer for 3 to 4 months (Osmocote^®^), formula NPK 15-09-12 with micronutrients: 0.0, 2.5, 5.0, 7.5 and 10 kg m^−3^.

When the seedlings presented the grafting pattern, between 4 and 5 mm in stem diameter, the experiment was set up to evaluate the compatibility between the black pepper plant ‘bragantina’ and the intra and interspecific rootstocks. The graft was obtained from cuttings collected from 2-year-old mother plants, in a commercial farm, located in the municipality of Linhares-ES located at 19°24′51″ S and 40°13′37″ W. The collected orthotropic branches were kept for 24 h in moistened burlap bags. The grafts were prepared with two nodes without leaves and the base prepared in the shape of a double-beveled wedge, measuring 3 cm. The full cleft top grafting method was used; grafting was carried out between 7:00 a.m. and 12:00 p.m.

The full cleft top forking method was carried out as described in detail in Albuquerque [32], with adaptations. The rootstocks were decapitated just below the fourth node, in which a perpendicular slit was made, with a depth of 3 cm, and after insertion of the graft, the graft was tied with rubber elastic number 18. Spraying was carried out with the fungicide Carbendazin and the graft was covered with 6 × 23 cm transparent plastic bags, being tied just below the graft, until the leaf was obtained, and the plastic bags were removed. After grafting, the plants were kept in a nursery with a 50% shade screen with sprinkler irrigation four times a day, with fungicide applied when necessary.

When the seedlings reached commercial standard (about four months after grafting), survival, chlorophyll *a* fluorescence, gas exchange, hydraulic conductivity of the stem and root, stem and root anatomy were assessed.

### 4.2. Chlorophyll a Fluorescence

At the end of the experiment, chlorophyll *a* fluorescence was evaluated in two plants per plot using the Hansatech portable fluorometer, model Handy-PEA (Hansatech, UK), in accordance with the recommendations of Strasser et al. [33]. Two leaves of each plant were adapted to the dark using leaf clips for 30 min, a period for complete oxidation of the photosystem. Afterwards, a saturating light flash of 3000 μmol m^−2^ s^−1^ photons lasting 1 s was emitted. From the OJIP transient fluorescence, the parameters established by the JIP Test were calculated. The interpretation and normalizations of the parameters measured and calculated from this test were in accordance with Strasser and Strasser [34].

### 4.3. Gas Exchange

At the end of the experiment, gas exchange was evaluated between 08:00 and 11:00 a.m. on fully expanded leaves located at the second or third node from the branch apex; two plants per plot were assessed. Analyses were performed using an infrared gas analyzer (IRGA), model LI-6400 (LI-COR, Lincoln, NE, USA), with a saturating photon irradiance of 1200 μmol m^−2^ s^−1^, a temperature of 25 °C, and a CO_2_ concentration of 400 ppm. Gas exchange assessments involved measuring net photosynthesis rate (PN) [μmol CO_2_ m^−2^ s^−1^], transpiration (E) [mmol H_2_O m^−2^ s^−1^], internal CO_2_ concentration (Ci) and stomatal conductance to water vapor (gs) [mol H_2_O m^−2^ s^−1^]. Based on these data, the following parameters were calculated: water use efficiency (WUE) (A/E) [(μmol m^−2^ s^−1^) (mmol H_2_O m^−2^ s^−1^)^−1^] and instantaneous carboxylation efficiency (EiC) (A/Ci) [(μmol m^−2^ s^−1^) (μmol mol^−1^)^−1^].

### 4.4. Anatomy

Anatomical assessments were carried out at the end of the experiment. Internode fragments from the stem of the rootstock and scion species and root fragments from the three rootstocks of three plants from each plot were collected. The tissues were fixed for 48 h in 70% FAA and after this time, they were stored in 70% alcohol. Transverse sections were carried out on the stem and root. Sections were cleared with sodium hypochlorite and stained with Safra blue. All sections were analyzed under a brightfield microscope Euromex (Duiven, The Netherlands). The images were captured with a microcamera CMEX 5 (Arnhem, The Netherlands) and tissue biometric measurements were performed with the ImageFocus 4 software.

Transverse sections were carried out on the stem in the rootstock species and in the cultivar used as a graft, where the vascular tissues in the cortex region were evaluated. Epidermis, vascular bundle width, vascular bundle length, xylem length, number of xylem vessels and diameter of xylem vessels were evaluated.

Regarding the roots, transverse sections were carried out in the root system of the species used as rootstock. The length of the vascular bundle and xylem, number and diameter of xylem vessels, cortex and vascular cylinder were evaluated.

### 4.5. Root and Stem Hydraulic Conductivity

The intact root system with substrate was immersed in water, subjected to pressures of 0.3 MPa, (60 s) inside a ‘Scholande’-type pressure chamber, model PWSC-3005, Soilmoisture E. Corporation (Goleta, CA, USA). The exudate was collected using absorbent paper with a defined mass and reweighed on a precision scale at each pressure applied.

In the second test, the method described by Liu et al. [35] and Li and Liu [36] was modified as recommended by Gambeta et al. [37]. The aerial part was sectioned on the branch emitted from the rooting cutting, 5 cm from it, with the stem immersed in water to prevent air from entering due to reflux at the time of cutting. An initial pressure of 0.03 MPa was applied so that the flow started for a minimum time of 3 min. Subsequently, the analysis pressures were applied respecting the minimum period of 3 min for flow stabilization, with the exudate being collected during the fourth minute of application of each pressure.

In both the root and the stem, the flow (J, kg s^−1^) was determined with the mass obtained as a function of the exudate collection time (60 s). The root hydraulic conductance (KR, kg s^−1^ MPa^−1^) was obtained by the relationship between the flow (J, kg s^−1^) and the pressure variation (ΔP, MPa) used. Root hydraulic conductivity was calculated by normalizing the KR value by dry mass (KR, RM, kg s^−1^ kg^−1^ MPa^−1^), root volume (KR, RV, kg s^−1^ L^−1^ MPa^−1^) and leaf area (KR, LA, kg s^−1^ m^−2^ MPa^−1^) [38].

### 4.6. Statistical Analysis

A randomized block design was employed, with four replications (blocks). For survival analyses, 108 plants per treatment were used (27 per block). For chlorophyll *a* fluorescence and gas exchange analyses, two leaves from two plants per block were used. For stem and root hydraulic conductivity and stem and root anatomy analyses, three plants from each block were used. In the case of treatments that showed low plant survival, statistical analyses were based on the actual number of surviving plants. The mean value for each characteristic was determined for each block, resulting in four means (replications). Statistical analyzes were carried out using the statistical program SISVAR version 4.3 [39]. After analysis of variance, the means were submitted to the Scott-Knott cluster test at a significance level of 5% (*p* < 0.05).

## 5. Conclusions

*P. aduncum* showed a reduction in the energy absorption flux per reaction center, energy captured flux per reaction center and energy dissipated flux per reaction center. The photosynthesis rate and stomatal conductance increased in *P. caldense* and bragantina plants.

Among the species studied, *P. aduncum* was the only one that showed significant differences in relation to the other species. *P. caldense* had the highest survival percentage and *P. aduncum* had the lowest survival.

Thus, it is concluded that, among the species studied, *P. caldense* was the species that exhibited the most favorable characteristics for early graft establishment and physiological performance as a rootstock when using the ‘bragantina’ genotype as the scion.

## Figures and Tables

**Table 1:** Photochemical parameters derived from the analysis of the JIP test on black pepper ‘bragantina’ grafted on different rootstocks, *Piper aduncum*, *Piper caldense* and bragantina. Means followed by the same letter in the column do not differ statistically from each other, by the Scott-Knott cluster test at 5% probability. Maximum photochemical quantum yield (φPo), quantum yield of electron transfer from QA to the electron transport chain beyond QA (φEo), photochemical quantum yield for heat dissipation (φDo), energy absorption flux per reaction center (ABS/RC), captured energy flow per reaction center (TR0/RC), dissipated energy flow per reaction center (DI0/RC), number of active FSII reaction centers per cross sections (RC/CS0) and performance index for energy conservation from captured excitons to the reduction of intersystem electron acceptors (PIabs).

Species	φ(P0)	φ(E0)	φ(D0)	ABS/RC	TR_0_/RC	DI_0_/RC	RC/CS_0_	PI_abs_
*Piper aduncum*	0.705 a	0.362 a	0.294 b	3.183 b	2.221 b	0.955 b	272.401 a	9.583 a
*Piper caldense*	0.637 b	0.255 b	0.362 a	3.985 a	2.494 a	1.490 a	234.551 b	3.861 b
bragantina	0.664 b	0.284 b	0.335 a	3.793 a	2.501 a	1.293 a	226.061 c	4.756 b

Means followed by the same letter in the column do not differ from each other according to the Scott-Knott test (*p* < 0.05).

## Data Availability

The original contributions presented in this study are included in the article. Further inquiries can be directed to the corresponding authors.

## Abbreviations

The following abbreviations are used in this manuscript:

ABS/RC	energy absorption flux per reaction center
DI0/RC	energy flux dissipated per reaction center
EiC	instantaneous carboxylation efficiency
gs	stomatal conductance
PIabs	performance index for energy conservation of photons absorbed by PSII for the reduction of intersystem electron acceptors
PN	photosynthetic rate
RC/CS0	number of active PSII reaction centers per cross section
TR0/RC	captured energy flow per reaction center
WUE	water use efficiency
φDo	photochemical quantum yield for heat dissipation
φEo	quantum yield of electron transfer from QA- to the electron transport chain beyond QA
φP_0_	maximum photochemical quantum yield
